# Providing crisis care in a pandemic: a virtual based crisis stabilization unit

**DOI:** 10.3389/frhs.2023.1030396

**Published:** 2023-05-16

**Authors:** Katrina Pullia, Avery Clavio, James M. Bolton, Erika Hunzinger, Sasha Svenne, Jennifer M. Hensel

**Affiliations:** ^1^ Department of Psychiatry, University of Manitoba, Winnipeg, MB, Canada; ^2^ Crisis Response Services, Shared Health, Winnipeg, MB, Canada; ^3^ Department of Psychology, University of Winnipeg, Winnipeg, MB, Canada

**Keywords:** virtual ward, virtual care, mental health, crisis care, COVID-19

## Abstract

**Background:**

Winnipeg, Canada operates a 16-bed subacute unit, the Crisis Stabilization Unit (CSU), for voluntary patients in crisis not requiring hospital admission. The virtual CSU (vCSU) launched in March 2020 as an adjunct to the in-person CSU during the COVID-19 pandemic, providing the same resources virtually, allowing patients to remain at home.

**Methods:**

Program data were collected for vCSU admissions between April 1, 2020 and April 7, 2021 (*n* = 266) to examine patient characteristics and discharge outcomes. Data were retrieved from the electronic patient record (EPR) for both in-person and vCSU admissions during the same period for comparison (*n* = 712). vCSU admissions (*n* = 191) were summarized by patient demographics, clinical factors/outcomes, and compared on the same measures to in-person CSU admissions (*n* = 521) using binary logistic regression.

**Results:**

30.1% of patients admitted to the vCSU received initial mental health assessment virtually (phone/videoconference), therefore receiving all care at home. Clinical symptoms at assessment included depression/anxiety (39.0%), psychosis/mania (2.7%), suicidal behaviour/self-harm (27.4%), psychosocial event/stressor (19.8%). Average stay was 4.9 days. Compared to the in-person CSU, vCSU referrals were associated with the absence of psychosis [odds ratio (OR).40, 95% confidence interval (CI).18–0.89] and no prior 1-year contact with referral site (OR.43, 95% CI.28–0.64). Those living farther away from the referral site were more likely to receive a vCSU referral.

**Conclusion:**

The vCSU model is feasible for a diverse group of patients experiencing mental health crises. Future work is needed to better determine who the model is right for and examine longer term outcomes.

## 1. Introduction

The COVID-19 pandemic created many challenges in the provision and accessibility of mental health crisis care. Due to the implementation of public health restrictions, changes in care delivery resulted in a reduction of in-person services and treatment (1). The reduction of these services may have disproportionately impacted the health of individuals living with mental illness (2). For example, individuals with mental illness were shown to be at increased risk of infection with COVID-19, and faced greater accessibility issues when seeking treatment (2). Additionally, pre-existing mental illness was associated with increased difficulty coping with the pandemic's effect on psychosocial factors leading to increased levels of stress, insomnia, depression, and anxiety compared to the general population (2–4). The combination of required changes to the administration of services due to public safety measures and the need for increased services for individuals with mental illness created a unique opportunity for the rapid expansion of virtual mental health services (5).

The concept of “virtual wards” was first introduced in the United Kingdom to provide short-term transitional home and community-based care for high risk and complex patients during their transition from hospital to their home and return to community (6). In Canada, virtual wards have been introduced in an attempt to reduce hospital re-admissions and their associated costs (7). The COVID-19 pandemic saw a proliferation of virtual wards that specifically leveraged technology to provide care in response to reductions in service capacity and the need to provide alternatives to in person visits (8, 9). For example, several virtual wards targeting the management of COVID-19 infection through the use of telephone and videoconferencing visits in combination with remote monitoring have been described (9).

In response to the decreased accessibility of in-person mental health services during the COVID-19 pandemic, Crisis Response Services in Winnipeg, Manitoba rapidly virtualized the delivery of a full spectrum of crisis care to individuals requiring urgent mental health services (10). Crisis Response Services is comprised of a 24/7 walk-in stepped care mental health crisis centre [the Crisis Response Centre (CRC); akin to a mental health-specific emergency department], a follow-up post-crisis care program, and the Crisis Stabilization Unit (CSU). The CSU is a community-based unit that provides short-term admissions and supportive care for voluntary individuals in crisis who do not require hospitalization (11). At the outset of COVID-19, the CSU reduced capacity by half to accommodate public health guidelines and staff were redeployed to open a 6-bed virtual CSU or “vCSU”.

The first part of this study aimed to describe the vCSU admissions, in terms of demographics, clinical characteristics, service delivery, and outcomes. The second part of the study involved a comparison of all referrals to both the virtual and in-person CSU from the largest referral source (the CRC) to identify factors that predicted the type of referral initiated. Given the novelty of virtual-based wards such as the vCSU, this study can inform the future planning and implementation of similar virtual models to increase the accessibility of mental health services.

## 2. Materials and methods

### 2.1. Study design

The first part of this study was a retrospective analysis of all admissions to the vCSU between the opening of the unit on April 1, 2020 and April 7, 2021 to examine patient characteristics and discharge outcomes. The second part was a secondary analysis of an administrative database consisting of all referrals to both the vCSU and in-person CSU from the largest referral source (CRC) from April 1, 2020 to April 7, 2021. Data were extracted from the electronic patient record (EPR) to compare the characteristics of the individuals referred to the different units.

### 2.2. Setting

The Winnipeg-based CSU is a 16-bed subacute unit for voluntary patients in crisis who may be at risk for hospitalization (11). Patients can be referred to the CSU from a variety of sources within the city including the CRC, emergency departments, urgent care centers, and specific community mental health services. The CSU is staffed by a multidisciplinary mental health team including crisis clinicians and nurses with access to psychiatry as needed. The CSU provides short-term supportive care and treatment with therapeutic group programing including skills-based classes as well as connection to community resources (11). Winnipeg is the largest urban centre in the province, home to just over 50% of Manitoba's total population (12). The majority of healthcare delivery is under provincial jurisdiction; CSU services are fully funded for all users.

**Figure 1:**
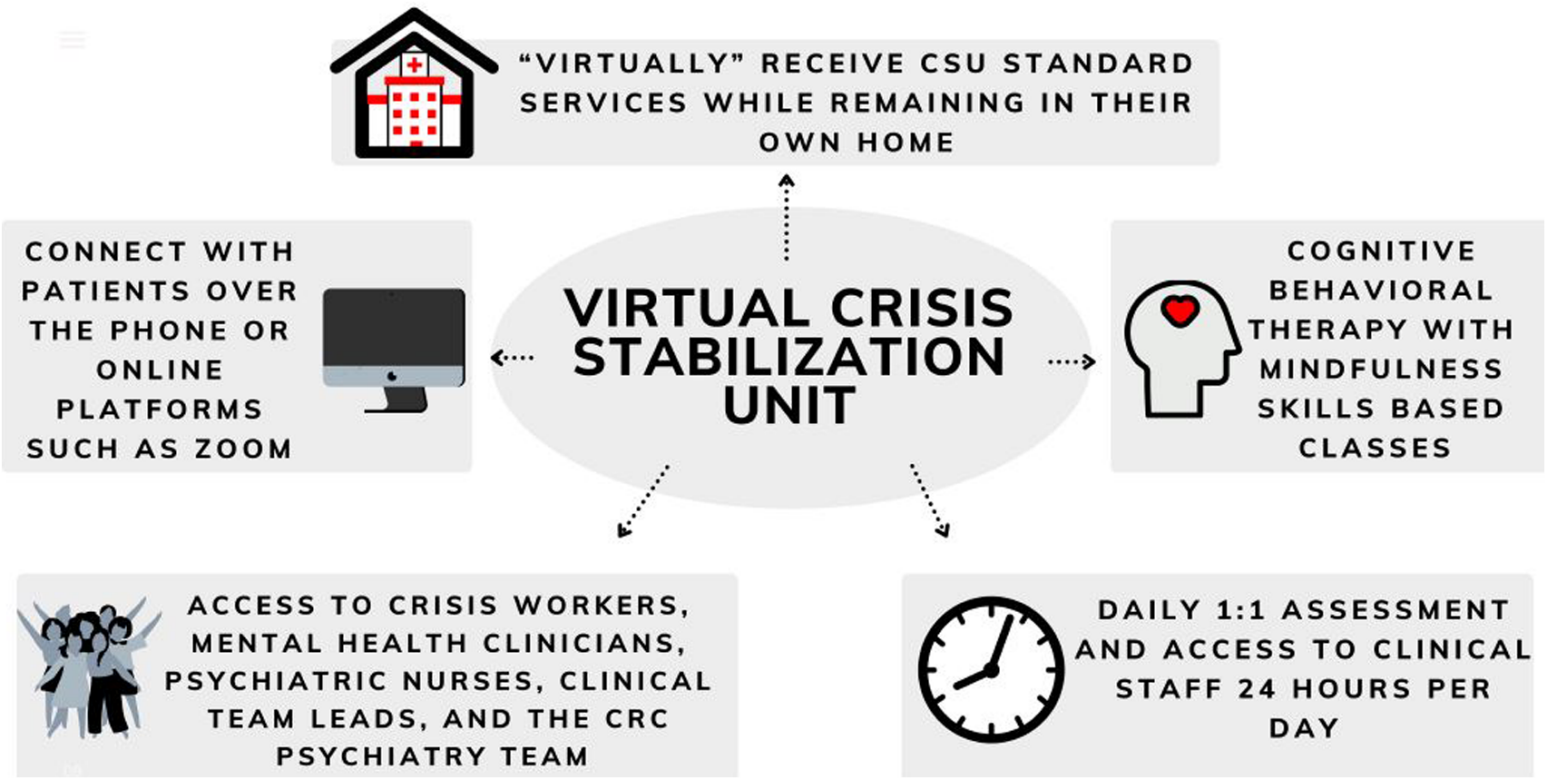
Summary of services offered by the virtual CSU.

The Virtual CSU (vCSU) was created as a full virtualization of the same services offered at the in-person CSU while permitting patients to remain in their own homes in the community. Services are delivered virtually by a combination of phone, text, email, and videoconferencing platforms (Zoom or Microsoft Teams) and patients have access to the same multidisciplinary staff as the in-person CSU including crisis workers, mental health clinicians, nurses, and psychiatry as needed. Patients are monitored daily and have access to clinical staff 24 hours per day for crisis support. They also have access to the skills-based classes available on the physical unit, delivered virtually, and are offered the same referrals and connections to community resources (Figure 1).

The same referral criteria applied for both units: the patient is voluntary, medically stable, not actively intoxicated or in acute withdrawal requiring medical intervention, and the patient is agreeable to commit to refraining from engaging in self-harm/behaviours and/or violence. A full mental health assessment and/or psychiatric consultation was required before referral describing the crisis episode which is interfering with functioning. There are no specific diagnostic exclusions or restrictions for patients except as it pertains to medical stability. Applicable to referrals to the vCSU only, the patient was required to have access to a technological device capable of accessing the service (either by phone or videoconferencing service) such as a mobile phone, tablet, or computer. The decision to refer to one or the other was based on clinical assessment and patient choice.

### 2.3. Participants/data sources

For all admissions to the vCSU, a vCSU clinician completed a form at the time of discharge that recorded patient demographics and clinical data as well as information about participation in vCSU services and outcomes. These forms were submitted to the study team approximately quarterly and inputted into a database by a research assistant.

For the second part of the study, an existing administrative database generated for another study (13) containing visit details extracted from the EPR at the CRC was used. The CRC represents the largest and most frequent source of referrals to both the CSU and vCSU accounting for over 70% of admissions. All individuals are identified by a unique CRC file number and all visits are assigned a unique visit number. The clinician or psychiatry team member conducting the mental health assessment enters sociodemographic and clinical data into the EPR which includes both fixed and free text fields.

### 2.4. Ethics

Research ethics approval for the study was obtained from the University of Manitoba Research Ethics Board [HS23878 (H2020:196)].

### 2.5. Variables

#### 2.5.1. Discharge forms

##### 2.5.1.1. Sociodemographics

Demographic data collected included age, gender (female, male, other) and living situation prior to admission (alone, with family, with roommates, other).

##### 2.5.1.2. Clinical information

Non-mutually exclusive categories were available to code reason for referral (medication management, risk/symptom monitoring, problem-solving/recovery planning, other) as well as main clinical problem at presentation (depression, anxiety, psychosis, mania, suicidal/self-harm, psychosocial event/stressor, personality disorder, other). The presence of suicidal ideation/self-harm was further characterized by the highest level identified at presentation (ideation, planning, or attempt). Origin of referral (CRC, Emergency Department, Urgent Care, Other) and active substance use (present or absent) were also captured.

##### 2.5.1.3. Service delivery

Variables assessing the delivery of vCSU service included length of stay, attendance for skills-based classes, method of contact with treatment team (phone or video), virtual family involvement for support and care planning while in the vCSU, and if psychiatry was consulted for an assessment during the stay.

##### 2.5.1.4. Outcomes

A variety of disposition options were available to patients after admission and were coded in non-mutually exclusive categories including discharge to pre-existing follow-up (primary care, psychiatry, other mental health supports), referral to the urgent post-crisis follow-up clinic, admission to hospital, detox/addiction services, or transfer to physical CSU. Some individuals were lost to follow-up prior to discharge.

#### 2.5.2. Electronic patient record (EPR)

##### 2.5.2.1. Visit type

The initial mental health assessment prior to referral could be conducted either in-person (individual seen onsite at the CRC) or virtual (assessment done by telephone or videoconference).

##### 2.5.2.2. Sociodemographics

Age at time of visit was calculated as the difference in years between visit date and date of birth. Gender was coded as male, female or other. Distance in kilometers (km) between the individual's residence and CRC was calculated using the CRC's address and geographic centre of the forward sortation area (FSA) corresponding to the first 3 digits of the individual's postal code. FSA data was obtained from Statistics Canada boundary files (14). As described elsewhere in previous work (13), we limited the sample to those living within 30 km of the CRC to reflect the typical geography of referral (Winnipeg and surrounding area) and to exclude individuals who had a recorded residence very far from the CRC and who may have been visiting Winnipeg or attending other services in the city prior to their CSU referral (n = 28 excluded). Median household income was retrieved from Statistics Canada's 2016 census profile for every FSA in the original dataset (15). A subset of the sample containing only unique individuals was created to generate income quintiles with Q1 representing the lowest household income and Q5 representing the highest. As a result, if individuals assessed at the CRC from specific income quintiles were more likely to be referred to the CSU, these will be overrepresented in the dataset.

##### 2.5.2.3. Clinical characteristics

For each CRC visit, a 1-year lookback was conducted to determine if the individual had a prior visit to the CRC within the last year. Diagnostic impression was based on the clinician assessment and available collateral information. Non-mutually exclusive categories included depression or anxiety (included adjustment problems, sleep problems, obsessive compulsive and trauma-related problems), psychosis, bipolar spectrum disorders, cognitive impairment (dementia, delirium, intellectual disability, acquired brain injury, autism), and other (ADHD, eating disorders and other impulse control problems). Presence of substance use was coded as present or absent.

### 2.6. Data analysis

For the first part of the study, descriptive statistics were generated for variables collected. All responses were calculated as counts and percentages or means as appropriate. For the second part of the study, descriptive statistics for the sample were generated, stratified by whether the referral was to the CSU or vCSU. To assess for relationships between the visit characteristics and the outcome (referral to the CSU or vCSU) we first conducted unadjusted binary logistic regressions for each variable using the visit as the unit of analysis, and then a fully adjusted model including all of the variables (age, gender, income quintile, distance, prior year visit, suicidal behaviour, substance use, and diagnostic impression). Model fit was evaluated using Hosmer and Lemeshow's goodness-of-fit test.

## 3. Results

### 3.1. Part 1: vCSU admission characteristics and outcomes

#### 3.1.1. Patient characteristics

Discharge forms were completed for a total of 266 patients who were admitted to the vCSU during the study period. Full data are summarized in Table 1. The average age of patients was 35 years old (range 18–77), the majority identified as female (*n* = 179, 67.3%) and most lived with family (*n* = 158, 59.4%). A wide variety of clinical symptoms were present at initial assessment including depression and anxiety (39.0%), psychosis or mania (2.7%), suicidal behaviour and/or self-harm (27.4%), and the presence of a psychosocial event or stressor (19.8%). Suicidal behaviour was further characterized by the highest level of severity present at assessment: ideation (53.0%), planning (12.4%), or attempt (7.5%). The reasons for referral to vCSU included medication monitoring (6.9%), risk and symptom monitoring (36.3%), problem solving and recovery planning (47.8%), and to initiate outpatient referrals (6.9%).

**Table 1:** Patient Variables from the vCSU Discharge Forms.

Baseline variable	Overall sample *N* = 266
Average Age [y], (range) [missing = 20]	34.8 (18–77)
Gender, *n* (%) [missing = 3]	
Female	179 (67.3)
Male	80 (30.1)
Other	4 (1.1)
Living situation, *n* (%) [missing = 9]	
Alone	60 (22.6)
With family	158 (59.4)
With friends/roommates	30 (11.3)
Other	9 (3.4)
Clinical symptoms present at initial assessment, *n* (%)^a^ [missing = 16]	
Depression & anxiety	244 (39.0)
Psychosis & mania	17 (2.7)
Suicidal behaviour and/or self-harm	171 (27.4)
Psychosocial event or stressor	124 (19.8)
Personality disorder	25 (4.0)
Other	44 (7.0)
Suicidal behaviour, *n* (%) [missing = 29]	
None	43 (16.2)
Ideation	141 (53.0)
Planning	33 (12.4)
Attempt	20 (7.5)
Active substance use, *n* (%) [missing = 4]	96 (36.1)
Reason for referral, *n* (%)^a^ [missing = 2]	
Medication management	31 (6.9)
Risk and/or symptom management	164 (36.3)
Problem solving and/or recovery planning	216 (47.8)
Referral to other sources	31 (6.9)
Other	10 (2.2)
Source of referral, *n* (%) [missing = 0]	
Crisis Response Centre	191 (71.8)
Emergency department or urgent care centre	70 (26.3)
Other	5 (1.9)
Referral assessment conducted virtually, *n* (%) [missing = 5]	80 (30.1)
Mean length of stay [days], (range) [missing = 10]	4.9 (1–10)
Assessed by psychiatry during vCSU stay, *n* (%) [missing = 27]	17 (6.4)
Attended minimum one skills class, *n* (%) [missing = 17]	102 (38.3)
Virtual family involvement during vCSU, *n* (%) [missing = 10]	15 (5.6)
Primary contact by phone, *n* (%) [missing = 19]	241 (90.6)
Discharge outcomes, *n* (%)^a^ [missing = 45]	
Pre-existing services (primary care, psychiatry, mental health)	191 (61.2)
Referral to outpatient mental health services	64 (20.5)
Detox or addictions services	5 (1.6)
Transfer to physical CSU	14 (6.0)
Admission to hospital	5 (1.9)
Lost to follow-up	7 (2.2)
Other	23 (7.4)

^a^ Non-mutually exclusive categories.

#### 3.1.2. Patient engagement

Nearly one third (30.1%) of initial assessments (mental health or psychiatry) that instigated the referral to the vCSU were conducted virtually with patients, meaning the patient never entered a facility. Analysis of service delivery variables revealed the primary means of communication with staff was over the phone (*n* = 241, 90.6%). Just over one third (*n* = 102, 38.3%) of all patients attended at least one virtual skills-based class during their stay. Psychiatry consultation occurred in 17 cases (6.4%). Similarly, 15 (5.6%) cases involved families or other supports in some way during the vCSU stay.

#### 3.1.3. Outcomes

There were a wide variety of discharge outcomes from the vCSU, most commonly to a pre-existing care team including primary care, mental health, or psychiatric services (61.2%). Twenty percent of patients were referred to outpatient mental health services. A small proportion of individuals were transferred to the in-person CSU (*n* = 14, 6.0%) and 5 individuals (1.9%) were admitted to hospital. Some reasons for transfer to the in-person CSU included: patient request, technological limitations, and clinician impression that the individual needed a higher level of observation and support. Reasons for hospital admissions included increasing self-harm and/or suicidal behaviour.

### 3.2. Part 2: CSU and vCSU comparison

#### 3.2.1. Patient demographics

A total of 712 visits to the CRC for 609 unique individuals during the study period resulted in referral to either the in-person CSU (*n* = 521) or vCSU (*n* = 191). Repeat admissions occurred for 9 of the vCSU admissions (4.7%) and 72 of the CSU admissions (13.8%). A small number of individuals had admissions to both units. The crude rates of sociodemographic factors did not appear to differ substantially between referrals resulting in admission to one or the other units, other than a higher proportion of vCSU referrals being present in the higher income quintiles. Level of suicidal behaviour was distributed almost equally between the groups. vCSU referrals had notably lower crude rates of prior year contact with the CRC than in-person CSU referrals (24.6% vs. 45.1%). Compared to CSU referrals, vCSU referrals had lower rates of substance use problems and psychosis, and higher rates of depressive and anxiety problems. Full results are summarized in Table 2.

**Table 2:** Rates of patient variables in CSU and vCSU cohorts.

Baseline variable	Overall sample *N* = 712	
	CSU (*N* = 521)	vCSU (*N* = 191)
Average Age [y] [missing = 0]	34.8	34.0
Gender, *n* (%) [missing = 5]		
Male	165 (31.8)	50 (26.6)
Female	343 (66.1)	136 (72.3)
Other	11 (2.1)	2 (1.1)
Income quintile, *n* (%) [missing = 1]		
Q1 – lowest	133 (25.6)	28 (14.7)
Q2	121 (23.3)	49 (25.7)
Q3	118 (22.7)	46 (24.1)
Q4	69 (13.3)	35 (18.3)
Q5 – highest	79 (15.2)	33 (17.3)
Suicidal behaviour, *n* (%) [missing = 9]		
None	136 (26.4)	50 (26.6)
Ideation	197 (38.3)	75 (39.9)
Planning	71 (13.8)	20 (10.6)
Self-harm/attempt	111 (21.6)	43 (22.9)
Had CRC assessment in prior year, *n* (%) [missing = 0]	235 (45.1)	47 (24.6)
Assessed by psychiatry at presentation, *n* (%) [missing = 0]	65 (12.5)	22 (11.5)
Substance use problem, *n* (%) [missing = 9]	232 (45.0)	70 (37.2)
Cognitive disorder, *n* (%) [missing = 9]	30 (5.8)	10 (5.3)
Depressive or anxiety problem, *n* (%) [missing = 9]	388 (75.3)	164 (87.2)
Personality disorder, *n* (%) [missing = 9]	183 (35.5)	62 (33.0)
Bipolar spectrum disorder, *n* (%) [missing = 9]	35 (6.8)	13 (6.9)
Psychosis, *n* (%) [missing = 9]	83 (16.1)	9 (4.8)
Other disorders (ADHD, eating disorder), *n* (%) [missing = 9]	35 (6.8)	12 (6.4)

9 cases missing data for suicidal ideation, substance use, diagnoses as seen by psychiatry only and these variables are not captured in that documentation.

#### 3.2.2. Referral analysis

The unadjusted and adjusted logistic regressions examining factors associated with referral to the vCSU relative to the in-person CSU are reported in Table 3. The fully adjusted model was a good fit for the data [*χ*^2^(8) = 5.31, *p* =.72]. In both the unadjusted and adjusted analyses, referrals to the vCSU were associated with living further away from the CRC [adjusted OR (aOR) 1.09 95% CI 1.03–1.15] and having no previous contact with the CRC in the past year (aOR.43 95% CI .28-0.64). In the unadjusted model, income quintile Q2, Q4 and Q5 were significantly more likely to be referred to the vCSU relative to Q1, the lowest. In the adjusted model, only Q2 remained significant. The absence of psychosis in the clinical assessment was significantly associated with a referral to the vCSU as opposed to the CSU, even after adjustment. While depressive and anxiety problems were significantly associated with referral to the vCSU in the unadjusted analysis, the correlation disappeared after adjusting for the other factors. The level of suicidal behaviour was not associated with referral outcome.

**Table 3:** Binary logistic regression for referral to the vCSU relative to the in-person CSU (*n* = 697).

Variable	Unadjusted		Adjusted	
	OR	95% CI	OR	95% CI
Age	.99	.98, 1.01	1.00	.98, 1.01
Gender (reference: female)				
Male	.76	.52, 1.12	.94	.62, 1.43
Other	.46	.10, 2.10	.38	.079, 1.80
Income quintile (reference: Q1, lowest)				
Q2	**1** **.** **87**	**1.10, 3.18**	**2**.**24**	**1.26, 3.97**
Q3	1.67	.97, 2.86	1.21	.69, 2.14
Q4	**2**.**29**	**1.28, 4.08**	1.28	.67, 2.45
Q5, highest	**1**.**99**	**1.12, 3.54**	1.29	.67, 2.49
Level of suicidal behaviour (reference: none)				
Ideation	1.05	.69, 1.61	1.14	.72, 1.80
Planning	.78	.43, 1.42	.90	.48, 1.69
Self-harm/attempt	1.05	.65, 1.70	1.14	.67, 1.94
Distance from residence to CRC	**1**.**07**	**1.03, 1.12**	**1**.**09**	**1.03, 1.15**
Prior 1-year visit (reference: no)	.**40**	**.27,.58**	.**43**	**.28,.64**
Substance use problem (reference: no)	.74	.52, 1.04	.79	.54, 1.17
Cognitive disorder (reference: absent)	.92	.44, 1.92	1.44	.63, 3.26
Depressive or anxiety problem (reference: absent)	**2**.**19**	**1.36, 3.52**	1.44	.80, 2.59
Personality disorder (reference: absent)	.90	.63, 1.28	1.03	.69, 1.54
Bipolar spectrum disorder (reference: absent)	1.06	.55, 2.06	1.19	.55, 2.59
Psychosis (reference: absent)	.**26**	**.13,.54**	.**40**	**.18,.89**
Other (reference: absent)	.94	.48, 1.86	.88	.43, 1.82

Bolded values represent statistically significant results (*P* < .05).

## 4. Discussion

The COVID-19 pandemic provided a unique opportunity for the rapid development of virtual mental health care services (5). The complete spectrum of mental health services offered through Winnipeg Crisis Response Services, including the vCSU, offered virtual options for services within weeks of the first documented COVID-19 case in Manitoba (15). Over 200 individuals with a wide range of mental health concerns at presentation, such as depression and anxiety, suicidal ideation, and psychosocial stressors, were able to access supports through the vCSU because of this transformation during a time of heightened restrictions, reductions in facility capacity, and high public anxiety. Patients admitted to the vCSU received the same clinical services as those admitted to the physical CSU and were connected to a variety of follow-up services as required. Our data demonstrate that a wide variety of patients presenting to a crisis centre can be referred to a virtual short stay crisis unit and managed in community.

Nearly one third (30.1%) of patients admitted to the vCSU were assessed entirely through virtual means from the outset of their presentation to crisis services and throughout their stay in the vCSU. This means that one third of all patients receiving these services did not have to enter a facility of any kind. The ability for patients to be assessed and receive services in their homes directly reduces the need for in-person assessments and in-person stays. This can not only relieve some of the burden on the healthcare system by limiting the use of inpatient beds, but it also has the potential to reduce problem of overcrowding and wait times in emergency departments and urgent care centres as patients can wait to receive assessments and services in their own homes (16).

Further, providing care to patients while allowing them to remain in the community has the potential to improve access to services for a multitude of patient-centered reasons. Patients often have competing demands such as work, school, and childcare that could hinder their ability to visit an in-person facility and wait an undetermined amount of time for an assessment (17). Even after receiving an in-person assessment, an extended in-person stay may not be a feasible option for someone with personal or family obligations. In comparison, a virtual ward admission would provide a similar level of support, however, allow for flexibility to continue attending to responsibilities in the home and community. For patients who live in rural areas or with limited access to public transportation, virtual care has the potential to improve accessibility and reduce costs associated with transportation and time (18). Although our analysis focused on referrals within Winnipeg and surrounding area, we still found that living further away from the CRC was associated with a referral to the virtual unit rather than the in-person CSU. There are a variety of patient-centered factors that influence care seeking which could be addressed with accessible virtual services. Virtual wards provide a convenient means of accessing intensive mental health supports in a stepped care fashion while continuing to remain in community and attend to personal commitments. A small proportion of individuals referred to the vCSU did ultimately require transfer to the physical CSU or admission to hospital and this could be co-ordinated directly from community.

Other factors that were associated with referral to the vCSU over the physical CSU were income and the absence of psychosis at presentation. Although all upper income quintiles were more often associated with a vCSU referral compared to the lowest income quintile in the unadjusted analysis, after adjusting for other factors, only Q2 remained significant in the model. This may point to the importance of some socioeconomic stability (eg. stable housing, availability of supports, employment, affordability of medications, and so on) in the lowest income groups to support a virtual stay, as opposed to the higher income groups where this may not be a differentiating concern. Given the clinical features of psychosis, it would be expected that in-person assessments and services would often be recommended for the treatment of this population (19). In addition, it is possible that this reflected patient preference and/or staff comfort with virtual services for individuals with these symptoms. No association, however, was found for a wide variety of other clinical symptoms, including the presence of suicidal behaviour. This suggests that there may be a subset of patients who present with suicidal behaviour who could be safely managed in community with access to virtual services and supportive interventions. The utilization of thorough suicide risk assessments to evaluate patients and their individual presentations would be an important component of this assessment and further research is warranted to identify additional factors that fit well with virtual care in the context of suicidal behaviour. In other work done by our team, some preliminary patient profiles that fit well with the model from the perspective of the providers were established to guide future service planning (20).

The predominant population admitted to the vCSU were adults who identified as female and who lived with family. The most common presenting issues at admission were depression and anxiety, suicidal behaviour/self harm, and/or the presence of a psychosocial event or stressor. The majority of referrals were initiated for risk and symptom monitoring or to assist with problem solving and recovery planning. In Manitoba, from 2014 to 2019, twenty-six percent of residents aged 10 or older were treated for at least one mental illness (21). The most common mental health presentations during this period were mood and anxiety disorders and the cumulative rate of mental illness for females was higher than males for all age groups (21). As such, the vCSU effectively captures a representation of the general Manitoba population who struggle with mental illness and are representative of the broader population receiving crisis support services.

### 4.1. Limitations

There are several limitations of our study. The vCSU was implemented rapidly during a unique situation created by the COVID-19 pandemic. The model remained stable throughout the study period, but as public health restrictions varied, patient preference and suitability for vCSU care may have shifted. For example, patients at high risk for or infected with COVID-19 were not eligible for in-person services. In the comparison analysis, data were only available for referrals from the CRC, and EPR data were not inclusive of other factors that could be influencing virtual admissions. Although there were repeat admissions for a portion of individuals, each visit was treated as an independent observation in the analysis since individuals can present for very different reasons from one visit to the next. There are, however, unmeasured variables that may affect referral to one or the other for a small number of frequent users of services (eg. care plans that limit number of admissions to the CSU in a given time period). Our study did not evaluate the perspective of the patients receiving these services and did not include a detailed examination of factors leading to transfer to in-person services. Future research would include input from patients especially regarding their preferences for care and the comparison of the perceived efficacy of virtually administered care compared to in person. While the data support that the vCSU increased access to mental health services during the COVID-19 pandemic, it is important to note that a lack of access to a phone or video conferencing technology device serves as a potential barrier to individuals for geographical and/or socioeconomic reasons that do not have, or cannot use, these communication platforms (22). This, along with other social determinants of health that are disproportionately experienced by those with lower socioeconomic status, speak to the complexities in understanding how to deliver virtual mental health services at a population level (23). This is an area requiring further exploration as systems move forward with expanded virtual care plans.

### 4.2. Conclusions

The unique situation created by the COVID-19 pandemic presented an unprecedented opportunity for the rapid establishment of novel virtual care models including the vCSU. This study has demonstrated this type of model as a safe, effective, and feasible mechanism for providing mental health crisis care. An admission to the vCSU can accommodate a wide variety of clinical presentations, provide services at an intensity that would otherwise be reserved for in-person facilities, and decrease wait times in emergency departments. Additional work is needed to further evaluate the effectiveness of virtual care compared to traditional in-person care and establish the sustainability and cost-effectiveness of virtual models of care. Virtual models can simultaneously provide person-centred care options and optimize health system resources and wait times.

## Data Availability

The original contributions presented in the study are included in the article/further inquiries can be directed to the corresponding author.
